# Correlates of prolonged television viewing time in older Japanese men and women

**DOI:** 10.1186/1471-2458-13-213

**Published:** 2013-03-09

**Authors:** Hiroyuki Kikuchi, Shigeru Inoue, Takemi Sugiyama, Neville Owen, Koichiro Oka, Teruichi Shimomitsu

**Affiliations:** 1 Department of Preventive Medicine and Public Health, Tokyo Medical University, Tokyo, Japan; 2 Behavioural Epidemiology Laboratory, Baker IDI Heart and Diabetes Institute, Melbourne, Australia; 3 Faculty of Sport Sciences, Waseda University, Tokorozawa, Japan; 4 School of Population Health, The University of Queensland, Brisbane, Australia; 5 Melbourne School of Population Health, The University of Melbourne, Melbourne, Australia; 6 School of Medicine, Monash University, Melbourne, Australia

**Keywords:** Sedentary behavior, Socio-demographic attributes, Population-based study, Cross-sectional study

## Abstract

**Background:**

In addition to insufficient moderate-to-vigorous physical activity (MVPA), prolonged sitting time is also a health risk for older adults. An understanding of population subgroups who have prolonged television viewing (TV) time, a predominant sedentary behavior, can aid in the development of relevant health promotion initiatives; however, few such studies have focused on older adults, the most sedentary segment of the population as a whole. The aim of this study is to examine the socio-demographic attributes associated with TV time among community-dwelling Japanese older men and women.

**Methods:**

A population-based, cross-sectional mail survey was used to collect data on TV time, MVPA, and socio-demographic characteristics. The survey was conducted from February through March 2010. Participants were 2700 community-dwelling older adults (aged 65–74 years, 50% men) who were randomly selected from the registry of residential addresses of three cities in Japan. Data from 1665 participants (mean age: 69.5 years, 52% men) who completed all variables for the present study were analyzed. Multivariate logistic regression analyses were used to calculate the odds ratios (ORs) of prolonged TV time (>2 hours/day) for each socio-demographic attribute, stratified by gender.

**Results:**

Of the 1665 participants, 810 (48.6%) watched TV for more than 2 hours/day. The median television viewing time (25th, 75th percentile) was 2.00 (1.07, 3.50) hours/day. Prolonged TV time was associated with not in full-time employment, lower educational attainment, weight status, living in regional areas and low MVPA for the whole sample. For men, prolonged TV time was associated with lower educational attainment; (OR = 1.53, 95% CI: 1.12-2.07), underweight (OR = 1.63, 95% CI: 1.02-2.60), overweight (OR = 1.57, 95% CI: 1.11-2.21), and low MVPA (OR = 1.43, 95% CI: 1.02-2.02). For women, living in regional areas (OR = 2.02, 95% CI: 1.33-3.08), living alone (OR = 1.61, 95% CI: 1.03-2.49), not driving (OR = 1.79, 95% CI 1.21-2.65), overweight (OR = 1.50, 95% CI: 1.00-2.24), and low MVPA (OR = 1.51. 95% CI: 1.05-2.17) were associated with prolonged TV time.

**Conclusions:**

These findings identify particular socio-demographic and behavioral characteristics related to TV time among Japanese older adults. It should be noted that correlates of prolonged TV time differed by gender. Women in living situations with limited transportation options tended to spend prolonged time watching TV. Health promotion initiatives for older adults, particularly for older women, may be more effective if they take these attributes into account.

## Background

Sedentary behaviors, which are distinct from lack of moderate-to-vigorous physical activity (MVPA), are associated with increased cardio-metabolic risk [1]. Studies have shown relationships between prolonged sitting and poor health outcomes independent of physical activity levels [2]. Television viewing (TV), a predominant sedentary behavior during leisure time, has an association with obesity and cardiovascular disease [3], atherosclerosis [4], the metabolic syndrome [5,6], and poor mental health [7]. Several longitudinal studies have found that prolonged TV time increases risk of type 2 diabetes [8] as well as all-cause and cardiovascular mortality [9]. Decreasing sedentary behavior, in addition to increasing physical activity, is now considered an important strategy to reduce health risk [10,11].

Older adults are the most sedentary segment of the population, potentially due to increased leisure time availability following retirement and declining functional capabilities [12,13]. TV viewing is a common leisure-time sedentary behavior among older adults [14], and older adults’ TV time can be associated with the prevalence of metabolic syndrome [15,16] and obesity [17]. However, little is known about which subgroups of older adults spend more time watching TV [15]. Such information is important in identifying specific population groups in need of future interventions. In the light of existing studies that show gender differences in demographic and behavioral correlates of sedentary behavior [14,18-20], we examined attributes associated with prolonged TV time among community-dwelling older men and women in Japan.

## Methods

### Participants and data collection

**Figure 1:**
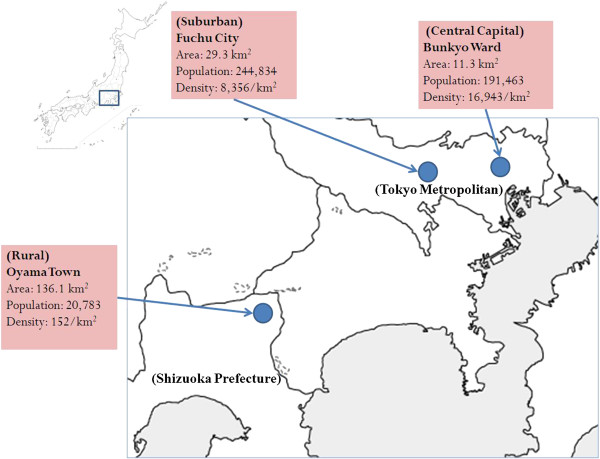
Location and characteristics of the three Japanese cities from which residents were recruited.

The study sample was recruited from 2700 community residents, from ages 65 to 74 years, living in three Japanese municipalities; Bunkyo Ward in Tokyo, Fuchu City in Tokyo, and Oyama Town in Shizuoka Prefecture (Figure 1). Bunkyo lies at the center of Tokyo (area: 11.3 km^2^, population: 191,463). Fuchu is a suburban city within the Tokyo Metropolitan Area located about 20 km east of the center of Tokyo (area: 29.3 km^2^, population: 244,834). Oyama is a small regional town, located about 80 km east of Tokyo (area: 136.1 km^2^, population: 20,783). In Japan, regional towns are typically less than 50,000 in population, and not in the vicinity of larger cities, such as a prefecture’s capital city. The sample was randomly selected from the registry of residential addresses of each municipality, stratified by gender (men, women), age (65–69 years, 70–74 years), and municipalities of residence (Bunkyo, Fuchu, Oyama); 1350 residents of each gender, 1350 residents of each age category, and 900 residents from each locality were identified. Data were collected from February to March 2010.

Potential participants received invitation letters that described the study. Two weeks after the invitation, they received a questionnaire and consent form. During the survey, a call center was set up to answer participants’ inquiries. To encourage participation, a 500-yen (about 5.5 U.S. dollars in 2010) book voucher was offered to respondents. For non-respondents, reminders to return the survey were mailed twice. Those who sent an incomplete survey were asked to complete the survey.

Of the 2700 initially identified, 2046 returned the survey. After data cleaning, the data from 1816 participants were deemed valid for this study (response rate: 67.3%). Among these respondents, 151 participants who had difficulty performing daily activities assessed by the Japanese 8-item Short-Form Health Survey (SF-8) [21] were excluded from the present analyses. The final sample size was 1665.

Participants signed an informed consent document before answering the questionnaire. This study received prior approval from the Tokyo Medical University Ethics Committee.

### Measures

#### Dependent variable

TV time was determined by participants’ self-reported frequency of watching television or videos (days/week) and average viewing duration per day (minutes/day) over the past 7 days. This question was translated into Japanese from an Australian questionnaire on leisure-time sedentary behaviors. The test-retest reliability of this questionnaire was reported as 0.82 [22]. In addition, significant associations with a three-day log (validity) were also reported [22]. Television viewing time is the particular sedentary behavior for which there is the strongest evidence of reliability and validity [23]. TV time was dichotomized based on the median value into two categories; short [≤2 hours/day] and long [>2 hours/day]. This cut point (>2 hours/day) was also reported as health risks in previous studies [5,24].

#### Independent variables

Age, gender and municipality of residence were obtained from the registry of residential addresses of each municipality. Living arrangements (living with others or living alone), educational attainment (years of education), employment status (working hours per week), self-rated health, dog ownership (having a dog, or not), driving status (driver, or non-driver), MVPA (minutes per week), body weight and height were obtained through self-report by each respondent. Self-rated health was assessed using an item from the SF-8: “Overall, how would you rate your health during the past 4 weeks?” Participants responded to the statement using a 6-point scale consisting of “excellent”, “very good”, “good”, “fair”, “poor”, and “very poor” [21]. For MVPA, the Japanese version of the International Physical Activity Questionnaire Short-version was used [25]. Participants were asked to report the frequency and duration of three types of physical activity: vigorous-intensity, moderate-intensity (excluding walking), and walking. Total time spent in MVPA including walking was calculated by adding these three activities together.

### Statistical analyses

Multivariate logistic regressions were employed to calculate odd ratios (ORs) and 95% confidence intervals (95% CI) of prolonged TV time (more than 2 hours per day) according to socio-demographic and individual variables. Physical activity was classified by tertiles: “low [<200 min/week], “medium [200–509 min/week]” and “high [≥510 min/week]”. This classification was used because a large proportion of participants (about 75%) reported 150 min/week or more MVPA, the current recommendation for older adults. Demographic variables were categorized as below; age (“65 to 69” or “70 to 74 years”), years of education (“up to high school [<13 years]” or “college degree or more [≥13 years]”), working hours (“none or part-time work [<35 hours/week]”, or “full-time work [≥35 hours/week]”), and self-rated health (“good” [excellent, very good, or good] or “poor” [fair, poor, or very poor]). Body mass index (BMI) was calculated from self-reported weight and height, and categorized into three categories; underweight (<20 kg/m^2^), normal weight (20–24.9 kg/m^2^), and overweight (≥25 kg/m^2^).

Analyses were conducted first for the overall sample, and then separately for men and women, adjusting for the demographic variables, MVPA, self-rated health, and BMI. All statistical analyses were performed using STATA software (version 10); the level of significance was set at p < 0.05.

## Results

Table 1 shows the characteristics of the participants. Men accounted for 52.0% of the respondents. The mean age of participants was 69.5 (sd: 3.0) years for men and 69.6 (sd: 2.9) years for women. The sample included about the same number of participants from each municipalities. The median TV time (25th, 75th percentile) was 2.00 (1.07, 3.50) hours/day overall, 2.00 (1.07, 3.46) hours/day in men and 2.00 (1.07, 3.50) hours/day in women, respectively.

**Table 1:** Characteristics of study participants

	**Total (n = 1665)**		**Men (n = 865)**		**Women (n = 800)**	
	**n**	**(%)**	**n**	**(%)**	**n**	**(%)**
Age group (years)						
65-69	827	(49.7)	430	(49.7)	397	(49.6)
70-74	838	(50.3)	435	(50.3)	403	(50.4)
Municipality						
Bunkyo (Urban)	540	(32.4)	283	(32.7)	257	(32.1)
Fuchu (Suburban)	567	(34.1)	300	(34.7)	267	(33.4)
Oyama (Regional)	558	(33.5)	282	(32.6)	276	(34.5)
Living arrangements						
Living with others	1479	(88.8)	782	(90.4)	697	(87.1)
Living alone	186	(11.2)	83	(9.6)	103	(12.9)
Educational attainment (education years)						
College degree or more (≥13)	606	(36.4)	389	(45.0)	217	(27.1)
Up to high school (<13)	1059	(63.6)	476	(55.0)	583	(72.9)
Employment status (working hours/week)						
Full-time work (≥35)	674	(40.5)	432	(49.9)	242	(30.3)
None or part-time work (<35)	991	(59.5)	433	(50.1)	558	(69.8)
BMI (kg/m^2^)						
<20.0	289	(17.4)	91	(10.5)	198	(24.8)
20.0 - 24.9	1040	(62.5)	577	(66.7)	463	(57.9)
≥25.0	336	(20.2)	197	(22.8)	139	(17.4)
Self-rated health						
Good	1401	(84.1)	734	(84.9)	667	(83.4)
Poor	264	(15.9)	131	(15.1)	133	(16.6)
Dog ownership						
Yes	231	(13.9)	128	(14.8)	103	(12.9)
No	1434	(86.1)	737	(85.2)	697	(87.1)
Driving status						
Yes	717	(43.1)	523	(60.5)	194	(24.3)
No	948	(56.9)	342	(39.5)	606	(75.8)
MVPA (minutes/week)						
<200	549	(33.0)	266	(30.8)	283	(35.4)
200 – 509	544	(32.7)	272	(31.4)	272	(34.0)
≥510	572	(34.4)	327	(37.8)	245	(30.6)
TV viewing(hours/day)						
≤2	855	(51.4)	457	(52.8)	398	(49.8)
>2	810	(48.6)	408	(47.2)	402	(50.3)

BMI: body mass index, MVPA: moderate to vigorous physical activity.

Table 2 shows the results of multivariate logistic regression analyses. For the whole sample; living in regional town, lower educational attainment, not in full-time employment (≥35 hours per week), underweight, overweight, and low MVPA (<200 min/week) were associated with longer TV time. In gender-specific analyses, men with lower educational attainment, not in full-time employment, underweight, overweight and low MVPA tended to watch TV longer. Among women, living in regional town, living alone, not in full-time employment, overweight, being a non-driver, and low MVPA were associated with prolonged TV time.

**Table 2:** Correlates of prolonged TV time by gender

	**Total**			**Men**			**Women**		
	**OR**	**(95% CI)**	**p**	**OR**	**(95% CI)**	**p**	**OR**	**(95% CI)**	**p**
Gender									
Women	1.00								
Men	1.21	(0.97 - 1.52)	0.098						
Age group (years)									
65-69	1.00			1.00			1.00		
70-74	0.89	(0.72 - 1.09)	0.257	0.96	(0.72 - 1.27)	0.764	0.81	(0.60 - 1.09)	0.164
Municipality									
Bunkyo (Urban)	1.00			1.00			1.00		
Fuchu (Suburban)	0.96	(0.75 - 1.23)	0.724	0.84	(0.59 - 1.20)	0.348	1.05	(0.73 - 1.49)	0.802
Oyama (Regional)	1.48	(1.11 - 1.97)	0.008	1.08	(0.72 - 1.63)	0.715	2.02	(1.33 - 3.08)	0.001
Living arrangements									
Living with others	1.00			1.00			1.00		
Living alone	1.26	(0.92 - 1.73)	0.156	1.01	(0.63 - 1.63)	0.963	1.61	(1.03 - 2.49)	0.035
Educational attainment (education years)									
College degree or more (≥13)	1.00			1.00			1.00		
Up to high school (<13)	1.37	(1.09 - 1.71)	0.006	1.53	(1.12 - 2.07)	0.007	1.24	(0.88 - 1.74)	0.224
Employment (hours/wk)									
Full-time work (≥35)	1.00			1.00			1.00		
None or part-time work (<35)	1.91	(1.54 - 2.36)	<0.001	2.20	(1.64 - 2.95)	<0.001	1.71	(1.24 - 2.37)	0.001
BMI (kg/m^2^)									
<20.0	1.35	(1.03 - 1.78)	0.031	1.63	(1.02 - 2.60)	0.041	1.24	(0.87 - 1.75)	0.228
20.0-24.9	1.00			1.00			1.00		
≥25.0	1.55	(1.20 - 2.01)	0.001	1.57	(1.11 - 2.21)	0.010	1.50	(1.00 - 2.24)	0.048
Self-reported health									
Good	1.00			1.00			1.00		
Poor	0.93	(0.70 - 1.22)	0.587	0.74	(0.50 - 1.10)	0.139	1.17	(0.79 - 1.73)	0.445
Dog ownership									
Yes	1.00			1.00			1.00		
No	0.95	(0.71 - 1.27)	0.722	1.08	(0.72 - 1.60)	0.721	0.80	(0.51 -1.25)	0.318
Driving status									
Yes	1.00			1.00			1.00		
No	1.27	(0.99 - 1.61)	0.057	1.01	(0.74 - 1.38)	0.959	1.79	(1.21 -2.65)	0.004
MVPA (min/wk)									
<200	1.46	(1.14 - 1.87)	0.003	1.43	(1.02 - 2.02)	0.040	1.51	(1.05 - 2.17)	0.028
200-509	1.24	(0.97 - 1.58)	0.088	1.27	(0.91 - 1.78)	0.167	1.25	(0.87 - 1.80)	0.223
≥510	1.00			1.00			1.00		

OR: odds ratio, 95% CI: 95% confidence interval, BMI: body mass index, MVPA: moderate to vigorous physical activity.

ORs were calculated by multivariate logistic regression analysis. All variables indicated in the table were included in the model.

## Discussion

The characteristics associated with prolonged TV viewing time (two or more hours a day) among older Japanese adults were: regional residence, living alone, lower educational attainment, not in full-time employment, underweight, overweight, and low MVPA. Older men and women differed in their correlates of prolonged TV time. The common correlates for men and women were: not in full-time employment, overweight, and low MVPA. As expected, prolonged TV time was more prevalent among older adults who were not in full-time employment and who presumably had more available leisure time. The lifestyle transitions associated with of retirement may be influential in this context. In previous studies, retirement, an important and common life-transition for older adults, has been identified as being associated with significant change in health-related behaviors such as smoking [26], alcohol consumption [27], and MVPA [28]. Retirement may be an important life transition stage in which to implement programs designed to reduce sedentary behaviors among older adults.

Among men, lower educational attainment was associated with prolonged TV time. Earlier studies have reported that educational attainment to have a strong association with various unhealthy behaviors among men, more than among women; this has been the case for smoking, alcohol consumption and low MVPA. Those with lower educational attainment may have less leisure-time options that compete with TV viewing.

For women, regional residence, living alone, and non-driving status were significant demographic correlates of prolonged TV time. These contextual factors may be related to the lack of convenient transportation options, which could hinder older women from going out. Since older women are less likely to have driver's licenses in Japan (84% in older men vs. 40% in older women) [29], those who live alone may have a limited means of transport, and tend to stay home for a long period. Similarly, those who live in regional areas have limited public transportation networks. For older adults, going outdoors is an important source of physical activity, and is associated with their functional and health status [30]. This study suggests that the lack of access to transport may pose a greater risk to older women’s health by hindering them from going outdoors and giving more time to watch TV indoors.

For men and women, being overweight (compared to normal weight) was associated with a higher likelihood of watching TV for a prolonged period. This is consistent with existing studies that have reported associations of prolonged TV viewing and obesity [31,32]. For men, there was an association of being underweight with prolonged TV time. Older men who are underweight, who may also be frail, may tend to sit for longer periods watching TV.

Our findings for older adults are consistent with some of the associations that have been observed for the correlates of television viewing time in young to middle-aged adults. This is the case in relation to lower educational attainment [5,14,24], living in a non-metropolitan area [14], overweight [5,7,24], and not being in paid work [14,24,33] being associated with more-prolonged TV time.

Although older age has been found to be associated consistently with TV time in previous studies [34], our findings identified no significant association with age categories. This is possibly due to the narrower age range of study participants. Regarding dog ownership, which was found to be a significant correlate in a previous study among Japanese young to middle aged adults [35], the present findings do not show a clear association. Possibly, those who were younger may have had less leisure time and thus need to sacrifice TV time to walk with their dogs. It may be that among older adults, dog ownership could act to increase MVPA, but may not necessarily decrease sedentary behaviors.

Some limitations and strengths of our study should be considered. First, all data were collected using self-report measures [36]. TV time is relatively accurate compared with other measures of sedentary behaviors [23], but it may have involved reporting bias. Second, a cross-sectional survey does not allow interpretations of the direction of causality. Reverse causality should be considered, especially for some variables such as BMI and self-rated health. Third, the age range of this sample was relatively narrow. The results of this study cannot be generalized to the older population (≥75 years). Studies including a wider range of older people are needed. Fourth, a relatively high proportion (75%) of our study participants reported levels of physical activity that could be classified as sufficient for health benefits. This may represent an over estimate by our participants, or may reflect some form of reporting or social-desirability bias. While this is a limitation that should be noted, the major focus of our study was not on physical activity per se. Finally, participant’s functional capacity was assessed using SF-8, an instrument to assess physical and mental health for adults in general. A more accurate measure of physical functioning is needed in future research. The strengths of this study include randomly-selected study participants from three different localities (urban, suburban, and regional).

## Conclusions

Prolonged TV time was associated with regional place of residence, living alone, lower educational attainment, non-full-time employment, weight status, non-driving status, and low MVPA among Japanese older adults. Employment status, weight status and MVPA were common correlates in both men and women. However, gender differences in the correlates of more-prolonged TV time were observed. Women’s TV viewing appears to be influenced by contextual factors such as location of residence, living with others, and non-driving status. Health promotion initiatives for older adults, and particularly for older women, may be more effective if they take these attributes into account.

## Competing interests

The authors declare that they have no competing interests.

## Authors’ contribution

SI, KO and TSI developed study design. HK, SI and KO collected data and conducted statistical analysis. HK and SI prepared draft version of the manuscript. All authors critically revised the manuscript for important intellectual content. All authors read and approved the final manuscript.

## Pre-publication history

The pre-publication history for this paper can be accessed here:

http://www.biomedcentral.com/1471-2458/13/213/prepub
